# Assessment of the Multi-Location External Workload Profile in the Most Common Movements in Basketball

**DOI:** 10.3390/s21103441

**Published:** 2021-05-15

**Authors:** Carlos D. Gómez-Carmona, Sebastián Feu, José Pino-Ortega, Sergio J. Ibáñez

**Affiliations:** 1 Research Group in Optimization of Training and Sports Performance (GOERD), University of Extremadura, 10005 Caceres, Spain; sibanez@unex.es; 2 BioVetMed & Sport Sci Research Group, Physical Activity and Sports Department, Sport Science Faculty, University of Murcia, 30720 San Javier, Spain; josepinoortega@um.es

**Keywords:** accelerometry, microtechnology, inertial devices, human body, impacts, team sports

## Abstract

The present study analyzed the multi-location external workload profile in basketball players using a previously validated test battery and compared the demands among anatomical locations. A basketball team comprising 13 semi-professional male players was evaluated in five tests (abilities/skills/tests): (a) aerobic, linear movement, 30-15 IFT; (b) lactic anaerobic, acceleration and deceleration, 16.25 m RSA (c) alactic anaerobic, curvilinear movement, 6.75 m arc (d) elastic, jump, Abalakov test (e) physical-conditioning, small-sided game, 10’ 3 vs.3 10 × 15 m. PlayerLoad_RT_ was evaluated at six anatomical locations simultaneously (interscapular line, lumbar region, knees and ankles) by six WIMU PRO^TM^ inertial devices attached to the player using an ad hoc integral suit. Statistical analysis was composed of an ANOVA of repeated measures and partial eta squared effect sizes. Significant differences among anatomical locations were found in all tests with higher values in the location nearer to ground contact (*p* < 0.01). However, differences between lower limb locations were only found in curvilinear movements, with a higher workload in the outside leg (*p* < 0.01). Additionally, high between-subject variability was found in team players, especially at lower limb locations. In conclusion, multi-location evaluation in sports movements will make it possible to establish an individual external workload profile and design specific strategies for training and injury prevention programs.

## 1. Introduction

There has been exponential development in technology for workload monitoring in indoor and outdoor conditions in basketball during the last few years [1]. First, the most extended methods to quantify workload were heart rate telemetry (Edwards, Training Impulse or Summated Heart Rate Zones) and rating of perceived exertion (RPE) from a physiological approach [2,3]. However, the monitoring of internal workload only provides information about the biological reaction of the human body produced by an unknown external workload [4]. So, time–motion analysis (TMA) was developed to monitor external workload during training and competition through video analysis or indoor radiofrequency technologies (e.g., local positioning system, ultra-wideband, etc.) [1]. Although TMA provided data about positioning, movements and their speed, these technologies underestimated the workload of high-intensity actions not involving locomotion (impacts, tackles, jumps, etc.) [5].

In this regard, microtechnology sensors (accelerometers, gyroscopes, magnetometers) have been developed for use in sports and to complete the data provided by TMA, integrating both in non-invasive units called inertial devices [1,2]. Specifically, the most used microsensor is the accelerometer, which records the changes in acceleration performed by the player, as a result of the interaction with gravitational forces and teammates/opponents in the three planes of movement [6].

The accelerometry-based demands during training and competition and the effect of contextual variables in basketball have been widely investigated in players and referees [6]. However, these demands were recorded with one device per player and at the scapular level, as this is accepted as the best location for TMA signal reception [7]. The problem is that accelerometers only record the acceleration of the body segment that they are attached to due to multi-joint complexity during sports movements [8]. So, the evaluation of external workload with different devices simultaneously and at different anatomical locations could provide information about how much external workload is progressively reduced by the natural absorption of the intermediate musculoskeletal structures between anatomical locations (scapulae, lumbar region, knee and ankle), as well as the lateral asymmetries between lower limb locations (left vs. right knee and ankle). The information could be useful for performance enhancement, injury prevention and return-to-play processes throughout the season, individually for each player and in general for the team [6].

For this purpose, a recent study proposed a field test battery to evaluate the individual multi-joint external workload profile in three invasion team sports (soccer, basketball and handball) [6]. This battery evaluates speed changes (accelerations and decelerations), changes in direction, jumps, and high-intensity and sprinting movements that are essential in basketball physical performance [9,10]. Therefore, the purposes of the present study were to: (a) characterize the multi-location external workload profile in the most common movements in basketball, (b) analyze the differences between the nearer anatomical locations (five segments: scapulae vs. lumbar region, lumbar region vs. right knee, lumbar region vs. left knee, right knee vs. right ankle, left knee vs. left ankle) to discern the vertical absorption of external workload by the musculoskeletal structures, and (c) identify the differences in external workload between anatomical locations of the lower limb (right knee vs. left knee, right ankle vs. left ankle) that could be associated with lateral asymmetries.

## 2. Materials and Methods

### 2.1. Design

A descriptive comparative design was followed to characterize the external multi-location workload profile in the most common movements in basketball and to analyze the differences in vertical musculoskeletal absorption and lateral asymmetries. No intervention was performed during the study, so it was given an ecological treatment [11].

### 2.2. Participants

A basketball team comprising 16 semi-professional male players was recruited in the present study. The evaluated participants belonged to a reserve basketball team of one that participates in the Spanish Federative First Division (LEB Oro League). The inclusion and exclusion criteria were: (a) absence of musculoskeletal injury or a health problem that impeded their participation in the testing, and (b) having experience of high-level monitoring by electronic performance tracking systems (EPTS) both in training and official games over more than two months [12]. As three players did not meet these criteria, the total sample was composed of 13 players (age: 19.48 ± 1.41 years; body mass: 87.63 ± 7.98 kg; height: 1.91 ± 0.07 m; body mass index (BMI, body mass divided by the square of the body height): 23.98 ± 1.45 kg/m^2^; muscle mass: 71.16 ± 5.79 kg; fat mass: 12.78 ± 3.22 kg).

Club managers, technical staff and players were previously informed about the investigation details and signed informed consent forms. The study was performed based on the ethical guidelines of the Declaration of Helsinki (2013) and approved by the Bioethics Committee of the University of Extremadura (registration number 232/2019).

### 2.3. Variables and Equipment

#### 2.3.1. Anthropometric Characteristics

Height, body mass, and BMI were assessed to characterize the participants in the study. Height was registered through a rod stadiometer (SECA, Hamburg, Germany) with 0.5 cm sensitivity and body mass and composition through an 8-electrode segmental monitor MC-780 MA model (TANITA, Tokyo, Japan).

#### 2.3.2. External Workload

Player Load by RealTrack Systems company (PL_RT_) was utilized to measure the external workload at the different body locations, obtained through WIMU PRO^TM^ inertial measurement units (RealTrack Systems, Almeria, Spain). These devices contain four 3D accelerometers (full-scale ranges: ±16 g, ±16 g, ±32 g and ±400 g), as well as other sensors (three 3D gyroscopes, a 3D magnetometer, a 10 Hz GPS, a 20 Hz UWB). Previous studies have shown the satisfactory reliability and accuracy results of the accelerometer in static and dynamic conditions [13]. The gyroscope and accelerometer were set with a sampling frequency of 100 Hz, the minimum recommended to record external workload in sport [14].

PL_RT_ is an accelerometer-derived measurement of total body load in its 3 axes (vertical, anterior-posterior and medial-lateral) that has been used to evaluate the neuromuscular load in different players [6]. This index calculates the vector sum of the four accelerometers that compose the inertial device, and it is represented in arbitrary units (a.u.). PL_RT_ is calculated from the following equation at a 100 Hz sampling frequency, where PL_n_ is the player load calculated in the current instant; n is the current instant in time; n-1 is the previous instant in time; X_n_, Y_n_ and Z_n_ are the values of body load for each axis of movement in the current instant in time; and X_n−1_, Y_n−1_ and Z_n−1_ are the values of body load for each axis of movement in the previous instant in time.

$$PL_{n}=\sqrt{\frac{{\left(X_{n}-X_{n-1}\right)}^{2}+{\left(Y_{n}-Y_{n-1}\right)}^{2}+{\left(Z_{n}-Z_{n-1}\right)}^{2}}{100}\;}$$

$$accumulated\;PL=\overset{m}{\underset{n=0}{\sum }}PL_{n}\times 0.01$$

The monitorization of PL_RT_ was performed by six inertial devices located in six anatomical locations simultaneously: (i) back (inter-scapulae line), (ii) lumbar zone (L3-L5, center of mass), (iii) knee (3 cm above the kneecap crack) and (iv) ankle (3 cm above the lateral malleolus) [13]. In the knees and ankles, the devices were placed on the outside of both legs. The players carried 0.5 kg extra (70–90 g for each of the six devices) during the testing. The attachment of the six devices to the player’s body was by means of a specific anatomically adapted one-piece sports vest (150–200 g) with two parts: (a) an upper body with two interior pockets to attach the back and lumbar devices, as well as an extensible band over the lumbar region to securely fix the device, and (b) a lower body with four exterior pockets with elastic bands to fix the devices on knees and ankles [15].

#### 2.3.3. Time Selection of Tests

Three hardware devices were used to perform the time selection in each test of each player in the timeline of the WIMU PRO^TM^ inertial devices. Firstly, a Windows tablet with SVIVO^TM^ software and with an Advanced and Adaptive Network Technology (Ant+) USB stick was checked to ensure the perfect functioning of the devices, as well as to mark the start and end point of each test. Ant+ is a wireless protocol for the collection and transfer of sensor data with an approximate range of 100 m. In addition to detecting the time when the players were running or jumping, Ant+ pushbuttons (RealTrack Systems, Almeria, Spain) and photocells (Chronojump, Barcelona, Spain) with Ant+ pushbuttons were used to send the data to the inertial devices with nearly perfect accuracy and reliability [16].

### 2.4. Procedures

The players’ assessments were carried out on their habitual court for training. The protocol was composed of four sessions. In the first session, the anthropometrical assessment (height, weight, and body composition) and the explanation of the study purposes were performed, while written informed consent was obtained by all study participants prior to the initiation of research. Then, familiarization sessions with high-level monitoring and with the battery of tests were administered during the second and third sessions. Finally, the following tests to evaluate the most common movements in basketball were performed in the fourth session.

(a) *Curvilinear movements:* Players ran at maximum speed around the 6.75 m line. Participants completed ten repetitions, where five repetitions were performed in each direction (left and right). When players finished each repetition, active rest of 1 min was taken. During the test, players had to run between the 6.75 m line and a line marked with cones at a distance of 1 m. If the participants fell or ran off the track, a new repetition was performed [15].
(b) *Jump capacity:* Players performed five jumps within the Abalakov test from the Bosco battery. This test consists of the execution of a countermovement jump with upswing of the arms [17]. Athletes started the test standing upright with the feet shoulder-width apart. Between jumps, there was a passive rest of 30 s.
(c) *Changes in speed:* Players performed five repetitions of the RSA test with a 16.25 m acceleration phase (from the free-throw line to the 6.75 m line) and a 5 m deceleration phase (from the 6.75 m line to the basket). Between repetitions, they performed active rest of 1 min. At the start, players had to place their feet behind the start line, and when the acceleration phase finished had to brake as soon as possible.
(d) *Linear movements:* Players performed the 30–15 IFT test, a standardized test both in distance and speed, adapted to a basketball court [18]. The test is composed of fractions with a 30-s run and a 15-s passive rest. Every 30 s, the speed is increased by 0.5 km/h. The test started at 8 km/h.
(e) *Game simulated conditions:* 10 min of a 3 vs. 3 small-sided game was played with official 3 vs. 3 rules on a reduced court with dimensions of 10 m × 15 m. To ensure compliance with the rules, an official referee participated in this test of the battery [15].

Previous to the data recording and following the manufacturer’s recommendations related to microelectromechanical sensors, three actions were performed: (1) turning on the device on a flat surface, (2) keeping it static for 30 s, and (3) ensuring there were no electromagnetic devices around it [13]. Additionally, the devices were turned on 1 h before assessment to achieve a constant temperature to ensure optimal data accuracy [19]. Then, the devices were attached using an anatomical vest to six anatomical locations simultaneously (scapulae, lumbar region, 2× knees and 2× ankles).

Players were cited 30 min before the testing to place the high-level monitoring systems. Twenty minutes before the start of the testing, a specific warm-up was performed that was composed of 10 min of moderate activity, 5 min of dynamic stretching and 3 min of light activity previous to the start of testing. Five min of between-test active recovery was performed.

### 2.5. Statistical Analysis

First, the data from the six inertial devices were downloaded. The software SPRO^TM^ was used to sync the data on the same timeline to be able to compare the recorded data during the same joint action and to calculate and export PL_RT_ data for each player in each test. Then, an exploratory analysis to determine the distribution and the homogeneity of data was carried out using the Shapiro–Wilk test and Levene test, respectively, showing a parametric distribution. So, a descriptive analysis (mean and standard deviation, M ± SD) was performed to characterize the sample.

An ANOVA of repeated-measures (one factor: 6 anatomical locations) with dataset segmented by tests was used to analyze the specific differences in the vertical (5 comparisons: (1) scapulae vs. lumbar region; (2) lumbar region vs. right knee; (3) lumbar region vs. left knee; (4) right knee vs. right ankle; (5) left knee vs. left ankle) and horizontal profile (2 comparisons: (1) left vs. right knee; (2) left vs. right ankle) in each type of movement independently. The post-hoc comparisons were analyzed with Bonferroni. The effect sizes were obtained by partial eta squared (*η_p_^2^*) and were interpreted as: *η_p_*^2^ < 0.01 trivial, *η_p_*^2^ = 0.01 to 0.06 low, *η_p_*^2^ = 0.06 to 0.14 moderate, and *η_p_*^2^ > 0.14 high [20]. The significance level was established at *p* < 0.05. Data analysis was performed with the Statistical Package for the Social Sciences (SPSS Statistics, version 24, IBM Corporation, Armonk, NY, USA) and figures (scatter dots plot where black line: mean; whiskers: standard deviation; dots: average value of each player) were designed with GraphPad Prism (Graphpad Ltd., version 8, La Jolla, CA, USA).

## 3. Results

### 3.1. Characterization of Multi-Location External Workload Profile in Male Basketball Players

**Figure 1:**
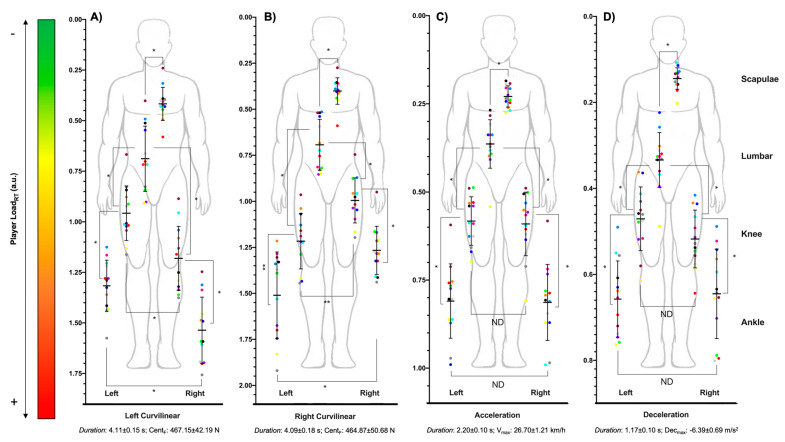
Scatter dots plot with mean (black line), whiskers (standard deviation) and dots (basketball players) to represent the multi-location external workload profile of semi-professional male basketball players in curvilinear movements ((**A**) left and (**B**) right direction) and speed changes ((**C**) acceleration and (**D**) deceleration). * Statistical differences (*p* < 0.05); ND: no statistical differences.

**Figure 2:**
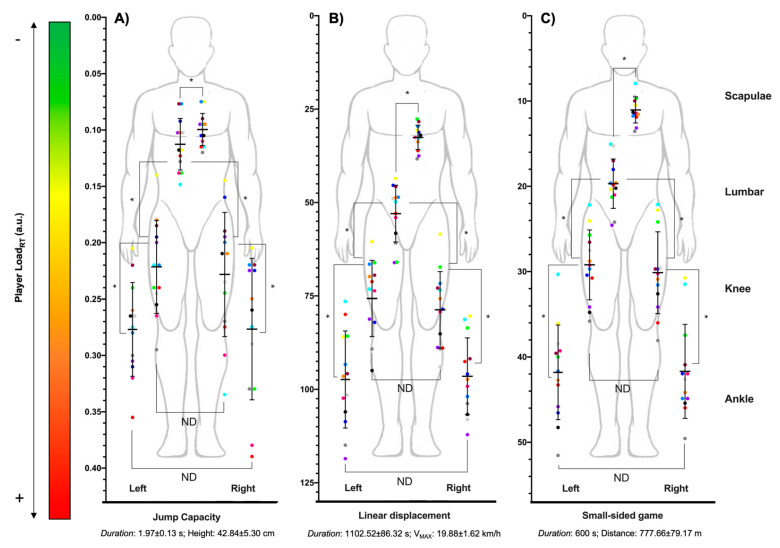
Scatter dots plot with mean (black line), whiskers (standard deviation) and dots (basketball players) to represent the multi-location external workload profile of semi-professional male basketball players in (**A**) jumps, (**B**) linear movements and (**C**) small-sided games. * Statistical differences (*p* < 0.05); ND: no statistical differences.

Figure 1 and Figure 2 show the multi-location external workload profile of each player in each test: jump (Figure 2A), linear running (Figure 2B), and game conditions (Figure 2C). Figure 1A,B shows the PL_RT_ presented in curvilinear movements (left vs. right direction) in scapulae (left: 0.42 ± 0.08; right: 0.40 ± 0.07), lumbar region (left: 0.69 ± 0.16; right: 0.69 ± 0.13), right knee (left: 1.18 ± 0.16; right: 1.00 ± 0.14), left knee (left: 0.96 ± 0.13; right: 1.20 ± 0.17), right ankle (left: 1.53 ± 0.16; right: 1.29 ± 0.18) and left ankle (left: 1.32 ± 0.13; right: 1.48 ± 0.23). Figure 1C,D show the external workload during changes in speed (acceleration vs. deceleration) in scapulae (acc: 0.23 ± 0.03; dec: 0.14 ± 0.02), lumbar region (acc: 0.36 ± 0.07; dec: 0.33 ± 0.06), right knee (acc: 0.59 ± 0.09; dec: 0.52 ± 0.07), left knee (acc: 0.58 ± 0.07; dec: 0.47 ± 0.07), right ankle (acc: 0.81 ± 0.11; dec: 0.64 ± 0.10) and left ankle (acc: 0.81 ± 0.11; dec: 0.66 ± 0.09).

Figure 2A shows the multi-location external workload profile during jumps (scapulae: 0.08 ± 0.01; lumbar region: 0.11 ± 0.02; right knee: 0.22 ± 0.06; left knee: 0.22 ± 0.05; right ankle: 0.27 ± 0.06; left ankle: 0.27 ± 0.05), Figure 2B during linear movements (scapulae: 32.67 ± 3.27; lumbar region: 53.02 ± 7.61; right knee: 78.79 ± 10.21; left knee: 75.68 ± 10.18; right ankle: 96.47 ± 10.29; left ankle: 97.35 ± 13.00), and Figure 2C during small-sided games (scapulae: 11.01 ± 1.53; lumbar region: 19.68 ± 2.89; right knee: 30.09 ± 4.80; left knee: 29.17 ± 4.09; right ankle: 41.67 ± 5.51; left ankle: 41.81 ± 5.55).

### 3.2. Differences between Anatomical Locations in Each Type of Movement

Table 1 shows the specific analysis of differences in each test between anatomical locations in the vertical and horizontal profiles through an ANOVA of repeated measures. Statistical differences were found between all anatomical locations in all tests (*F =* 87.80 to 333.33; *p* < 0.01; *η_p_^2^* = 0.86 to 0.97 high effect). Specifically, in the vertical profile, differences were found between all anatomical locations with higher values in the location nearer to ground contact in all tests (*p* < 0.01), except in jumps where player 2 presented higher values in the right knee in comparison with the right ankle.

Regarding the horizontal profile, differences were found in left curvilinear movements at the knee and ankle, with higher values in the right leg in all participants (*p* < 0.01), and in right curvilinear movements at the knee and ankle, with higher values in the left leg (*p* < 0.01), except in players 10 and 12, with higher values in the right ankle. However, no horizontal differences in the knee and ankle were found in accelerations (*p* = 1.00), decelerations (*p* > 0.32), jumps (*p* = 1.00), linear movements (*p* = 1.00), and small-sided games (*p* = 1.00).

## 4. Discussion

Most of the investigations into monitoring the external load using accelerometry in team sports for load quantification are performed in a single anatomical location, preferably the scapulae, due to the better reception of the tracking position in indoor or outdoor conditions [6]. The assessment of sports performance in basketball has been performed in physical fitness tests (endurance, power, strength and agility) [21] and during competitive situations [3]. These evaluations can obtain the distance covered, the time spent, the speed reached, or the force generated as a performance index. Accelerometers only detect the load on the location or segment to which they are attached [8]. In these traditional evaluations, the problem is that they only considered the total load recorded in one anatomical location, but not how the load is supported by the musculoskeletal structures, as well as the possible asymmetries in the lower limbs individually [22]. The behavior of neuromuscular load throughout the human body is specific concerning the volume and intensity of movements, as well as the anatomical location [6]. Due to the association between neuromuscular load and injury risk, the study of the accelerometry-based workload can provide useful information for the individualization of training programs [8,23]. Therefore, the purpose of this study was to characterize the multi-location external workload profile in semi-professional basketball players during the most common movements in basketball and analyze the vertical (scapulae vs. lumbar region, lumbar region vs. right knee, lumbar region vs. left knee, right knee vs. right ankle, left knee vs. left ankle) and horizontal differences (left vs. right knee, left vs. right ankle). Statistical differences were found in the vertical profile (*p* < 0.01), with the highest workload in the ankle and a progressive decrease in external workload as the distance of the joint from the ground increases. Regarding laterality, differences were found only in curvilinear movements (*p* < 0.01) related to their direction, with a higher impact on the outside leg.

Currently, every detail should be considered at high performance, and the monitoring of the external load in different locations simultaneously could provide more reliable and valid information of the total load that the player supports [6]. From this assessment, individual profiles could be detected in musculoskeletal structures. The study of individual variability is essential to adapt the training load and achieve the desired stimuli for performance enhancement [24], as shown in the results of the present study. Regarding the vertical profile, all players suffered a higher external workload in the location nearer to the ground contact in comparison with the further locations as found in previous research [8,23], although the percentage of differences between the upper and lower location is variable among subjects. However, in the horizontal profile, great variability in the accelerometry-based workload was found in the lower limb (ankle, knee), where the highest number of non-contact injuries occur in team sports [25]. This variability was found in acceleration, deceleration, jumps and linear movements, not finding predominant laterality in team players. Therefore, the detection of individual profiles seems to be fundamental for designing specific training and injury prevention sessions with the aim of maintaining the optimal physical level for a longer time [26]. Strategies such as running gait programs and unilateral strength (concentric-eccentric-isometric) training could be considered to improve these deficiencies [27,28]

The external workload profile is not only affected by the physical fitness and the individual characteristics of each player; the intensity, volume and direction of movements have a determinant role in the neuromuscular workload dynamics concerning the propulsive and braking forces against the ground [22]. Numerous studies have identified a high variability in musculoskeletal activation through electromyography [29], in psycho-physiological response through heart rate, rating of perceived exertion and wellness [22], as well as in sports performance through time, distance covered, speed or generated force during movements in basketball [3]. All these factors are going to have a direct effect on the impacts suffered by the players’ musculoskeletal structures. In the present study, the highest impact differences between the scapulae and lumbar region were found in the deceleration phase, between the lumbar region and knees in jumps, and between the knees and ankles in the acceleration phase and small-sided games. As mentioned above, different investigations have found a key role of the core in the deceleration phase [30], greater muscular activation of the thigh in jumping [31] and a high load and involvement of the calves and soleus in the acceleration phase [32]. This aspect is especially accentuated in basketball, where speed changes (accelerations and decelerations) are frequently performed due to the temporal limitation of playing actions and the reduced court space [33].

Another very important aspect to be considered is curvilinear movement. This type of movement plays an important role in the game, since specific actions such as receiving the ball, blocking continuations, blocking exits or reversals require a high level of curvilinear movements and changes in direction, being essential in the physical performance of basketball [9,10]. During curvilinear movements, the centripetal and centrifugal force must be added to horizontal and vertical forces suffered during linear movements [34]. For this reason, a greater external load was found on the outside leg compared to the inside leg, both in the ankle and the knee depending on the direction of movement (*p* < 0.01). This causes an imbalance between the force exerted by each leg during movements. The role of the inside (pivot point and to help the impulse) and outside leg (to maintain the player in the curvature and with a determinant role in impulse) is different, so that optimal levels of balance and body control are necessary [35,36]. Therefore, specific work must not only be carried out according to the individual characteristics but also concerning the type, volume and intensity of movements that are specific according to their role and the level of competition during official games [26]. The identification of these possible anomalies in the external workload absorption by the musculoskeletal structures may imply the re-education of the motor pattern in each type of movement, as well as the adaptation of the training workload to continue the process of performance improvement, decreasing the injury risk.

In addition to the assessment of sports performance, the multi-location profile could provide useful information for injury prevention. Injury is multifactorial with non-modifiable and modifiable risk factors [37]. Intervention on modifiable risk factors (e.g., strength, biomechanics, flexibility, balance, etc.) through a systematic process may reduce the injury risk [25]. The multi-location external workload assessment will provide useful information to create individual musculoskeletal absorption profiles and to consider them to check the evolution of the players throughout the season as well as whether the training process has the desired effect on each muscle segment or joint [6,8,22,23]. Due to the between-subject differences related to anthropometrics, laterality, body composition, playing position and specific role, as well as the history of previous injuries, it is advisable to identify individual profiles [38], thus making it possible to adapt and individualize the training workload, both total and specific, in each anatomical location.

## 5. Limitations and Future Research

While the results of this study provide the first approach to the multi-location external workload profile through six inertial devices simultaneously attached to the player in an integral vest, and differences between anatomical locations and between players were found to identify asymmetries and vertical impact absorption through musculoskeletal structures in the most common movements in basketball, some limitations to the study must be acknowledged. The main limitation in this research concerns the sample studied (13 basketball players at a semiprofessional level), although the application of a previously validated multi-location field test battery [15] has been shown to be effective for detecting the external workload in different body locations simultaneously, and creating individual profiles. It is important to consider that the data obtained can only be extrapolated to players with the same individual characteristics and competitive level. In addition, 36 inertial devices were necessary to evaluate all the players simultaneously in the same conditions, so it is recommended to collaborate with research teams for the evaluation and counseling of players at all competitive levels.

Finally, future research could use this assessment method to analyze the external load supported by different anatomical locations, and based on a larger population, identify if the percentage of difference in the external load between anatomical locations represents a pathology and design specific interventions for each of them.

## 6. Conclusions

From the evaluation of the multi-location external workload profile of basketball players in the most common movements in basketball, different conclusions and practical applications can be provided:

### 6.1. Vertical Profile

1. All players presented a higher workload in the anatomical location nearer to the ground contact in comparison with the further locations. The highest external workload was found in the lower limb (ankle and knee). Team staff should consider more extensive recovery protocols in the lower body to alleviate the workload suffered during training sessions and official games by these musculoskeletal structures.
2. The greatest variability of external workload was found in the lower limb in comparison to the upper limb. The design of training sessions must be individualized according to the musculoskeletal profile and the individual characteristics of each player (laterality, flexibility, strength and previous injuries), with special consideration for the lower limb.
3. The greatest differences between the scapulae and lumbar region were found during the deceleration phase, between the lumbar region and knee in jump actions, and between the knee and ankle in the acceleration phase and small-sided games. The identification of how the musculoskeletal structures support the external load in each type of movement will help team staff to detect movement patterns that may be specifically trainable.

### 6.2. Horizontal Profile

1. Differences in laterality between the knees and ankles were found in curvilinear movements. The highest workload was found in the knee and ankle of the outside leg in comparison with the inside leg. The specific training of actions that involve curvilinear movements and changes in direction at high intensity in basketball will help in the improvement of players’ performance and injury prevention, especially due to the different motor patterns of each leg according to the direction of movement.
2. However, no differences in the external workload suffered by the knees and ankles were found in acceleration, deceleration, jump, linear movement and small-sided games. Therefore, the training of the lower limb must be completed according to the type of movement.

## Figures and Tables

**Table 1:** Differences in the multi-location external workload profile in the most common movements in basketball.

Test	ANOVA of Repeated Measures			Bonferroni Post Hoc	
	*F*	*p*	*η_p_^2^*	Vertical Profile	Horizontal Profile
Left curvilinear	225.88	<0.01	0.95	a b c d e	f g
Right curvilinear	175.56	<0.01	0.93	a b c d e	f g
Acceleration	214.76	<0.01	0.95	a b c d e	
Deceleration	171.38	<0.01	0.94	a b c d e	
Jump	87.80	<0.01	0.88	a b c d e	
Linear	186.53	<0.01	0.94	a b c d e	
Small-sided game	333.33	<0.01	0.97	a b c d e	

Note. F: F-value of repeated-measures ANOVA; *p*: significance of repeated-measures ANOVA; *η_p_*^2^: partial eta squared. a: Statistical differences between scapulae and lumbar. b: Statistical differences between lumbar and right knee. c: Statistical differences between lumbar and left knee. d: Statistical differences between right knee and right ankle. e: Statistical differences between left knee and left ankle. f: Statistical differences between right knee and left knee. g: Statistical differences between right ankle and left ankle.

## Data Availability

The data presented in this study are available on request from the corresponding author. The data are not publicly available as the Organic Law 3/2018, of 5 December, on the Protection of Personal Data and Guarantee of Digital Rights of the Government of Spain requires that this information must be in custody.
